# Inter-Frame-Relationship Protected Signal: A New Design for Radio Frequency Fingerprint Authentication

**DOI:** 10.3390/s23156948

**Published:** 2023-08-04

**Authors:** Xufei Li, Shuiguang Zeng, Yangyang Liu

**Affiliations:** 1 School of Computer Science and Technology, Xidian University, Xi’an 710071, China; liuyangyang@stu.xidian.edu.cn; 2 College of Computer and Cyber Security, Hebei Normal University, Shijiazhuang 050024, China; sgzeng@hebtu.edu.cn

**Keywords:** radio frequency fingerprint authentication, CSMA/CA, inter-frame-relationship, carrier frequency offset

## Abstract

Utilizing a multi-frame signal (MFS) rather than a single-frame signal (SFS) for radio frequency fingerprint authentication (RFFA) shows the advantage of higher accuracy. However, previous studies have often overlooked the associated security threats in MFS-based RFFA. In this paper, we focus on the carrier-sense multiple access with collision avoidance channel and identify a potential security threat, in that an attacker may inject a forged frame into valid traffic, making it more likely to be accepted alongside legitimate frames. To counter such a security threat, we propose an innovative design called the inter-frame-relationship protected signal (IfrPS), which enables the receiver to determine whether two consecutively received frames originate from the same transmitter to safeguard the MFS-based RFFA. To demonstrate the applicability of our proposition, we analyze and numerically evaluate two important properties: its impact on message demodulation and the accuracy gain in IfrPS-aided, MFS-based RFFA compared with the SFS-based RFFA. Our results show that the proposed scheme has a minimal impact of only −0.5 dB on message demodulation, while achieving up to 5 dB gain for RFFA accuracy.

## 1. Introduction

Radio frequency fingerprint authentication (RFFA) is a novel approach that leverages the inherent randomness of radio frequency hardware imperfections to authenticate transmitters. These hardware imperfections, including carrier frequency offset (CFO) [1], in-phase/quadrature (I/Q) imbalance [2], and I/Q origin offset [3], possess inherent, unique, and non-reproducible properties. Thus, they can be used for identity authentication without the need for traditional credentials such as tokens or digital signatures. As a result, RFFA has emerged as a prominent technology for identity authentication in future wireless networks [4,5,6].

Although RFFA has been extensively studied over the past few decades, achieving high accuracy remains a significant challenge. To address this issue, many researchers have devoted themselves to two approaches. The first approach is to explore potential hand-crafted features according to underlying hardware imperfections. For instance, the authors in [7] first proposed five features, and the authors in [6] proposed a new feature called fractal dimension that can be used for RFFA. The second approach is to utilize machine learning techniques to automatically extract and apply the features for RFFA. For example, the authors in [8] proposed a machine learning-based method to dynamically determine the feature decision threshold in RFFA, and the authors in [9] proposed an incremental learning method to continuously realize the feature extraction. The complicated computation involved in the second approach has also been of concern; for example, the authors in [10] proposed a transfer learning method to reduce the computation required for edge nodes while accurately extracting the feature. Note that, for both of these two approaches, applying a multi-frame signal (MFS) performs with higher accuracy than a single-frame signal (SFS) as the input of the authenticator. This is because the MFS-based RFFA leverages the integration of multiple frames to mitigate the adverse noise effect. This approach is practical to implement, since one communication session typically involves multiple frames serving as candidates for constructing the MFS. As a brilliant study, the authors in [7] demonstrated that, by increasing the number of frames involved in the signal from 1 to 10, the RFFA accuracy improved from 30% to 90%. Despite the benefits touted by many researchers regarding this approach, they have frequently overlooked the potential security threats associated with it.

**Figure 1:**
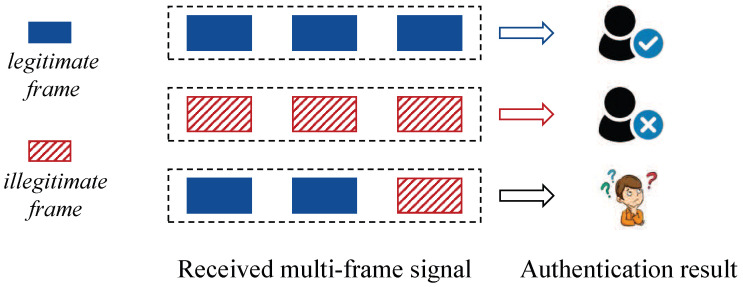
Three traffic types of received frames: all frames are legitimate, all frames are illegitimate, and some frames are legitimate while the rest are illegitimate. Each type of traffic is regarded as a whole in MFS-based RFFA.

In this paper, we address a security threat associated with the aforementioned approach. Our observation is that, if an attacker injects a forged frame into the valid traffic, the forged frame can potentially blend in with other legitimate frames during the authentication process, as illustrated in Figure 1. Intuitively speaking, this may increase the likelihood of the forged frame being accepted, in a way that is even more pronounced compared with that of the single-frame signal (SFS)-based RFFA. To validate this intuition, we also conducted a Proof-of-Concept experiment (see Section 3.3), and the results clearly demonstrated this security threat. Regrettably, conducting such an injection attack is relatively straightforward for carrier-sense multiple access with collision avoidance (CSMA/CA). This is because that attackers can arbitrarily employ an idle channel to conduct the traffic injection by modifying the Backoff time [11]. Given the widespread application of CSMA/CA, it becomes crucial to address this security threat when promoting the adoption of MFS-based RFFA.

To provide an MFS-based RFFA scheme overriding the above security threat, we propose an innovative design called the inter-frame-relationship protected signal (IfrPS). The core concept of IfrPS is to bind each pair of consecutively transmitted frames’ signal with unique information, which can be used by the receiver to determine whether two consecutively received frames originate from the same transmitter. Meanwhile, frames that do not conform to the inter-frame relationship are excluded from the MFS-based RFFA process. Note that the unique information is randomly generated for each pair of consecutively transmitted frames and, thus, it cannot be forged by the attacker. Note that the proposed IfrPS-aided, MFS-based RFFA is applicable to these CSMA/CA communication systems, such as IEEE 802.15.4 and IEEE 802.11, in which a security level is required.

To demonstrate the applicability of our proposition, we considered two properties: efficiency and effectiveness. Efficiency evaluates the impact of IfrPS on message communication, while effectiveness quantifies the accuracy improvement achieved by IfrPS-aided, MFS-based RFFA compared to an SFS-based one. The main contributions of this paper are summarized as follows:

1. This study is the first to identify a security threat associated with MFS-based RFFA. In the CSMA/CA scenario, an attacker can inject forged frames into legitimate traffic. The MFS-based RFFA would be compromised when such an injection is not detected. We further substantiate this security threat through a Proof-of-Concept experiment;
2. To address this security threat and provide a robust MFS-based RFFA scheme, we propose the IfrPS design. The designed IfrPS can be integrated into the MFS-based RFFA to enable the receiver to detect injected frames within valid traffic. Moreover, IfrPS requires no pre-shared key between the transceiver and is compatible with old receivers because it does not need to authenticate the transmitter;
3. We analyze the potential impact of the IfrPS design on message demodulation for different constellations, including BPSK, QPSK, and 16QAM. Theoretical analysis and numerical evaluations demonstrate that the IfrPS design causes minimal degradation to message demodulation, with approximately −0.5 dB observed for the BPSK modulation system;
4. To quantify the accuracy improvement in the IfrPS-aided, MFS-based RFFA compared with the SFS-based one, we conducted a case study using CFO as the authentication feature. Through theoretical analysis and numerical evaluations, we assessed the false reject ratio (FRR) at different false accept ratio (FAR) levels. The results indicate that the proposed approach can achieve up to 5 dB gain compared to the SFS-based RFFA.

The remainder of this paper is organized as follows. Related work is introduced in Section 2. Section 3 presents the system model and security threat. In Section 4, we present the designed IfrPS and the IfrPS-aided, MFS-based RFFA scheme. In Section 5, we study the efficiency of the IfrPS design, and, in Section 6, we study the effectiveness of the IfrPS-aided, MFS-based RFFA. This paper is concluded in Section 7.

The abbreviations used in this paper are summarized in Table 1.

## 2. Background

### 2.1. RFFA

As listed in Table 2, there are two approaches in RFFA research. To the best of our knowledge, [7] is the first approach for exploiting hardware imperfections to serve as the wireless device identity for RFFA. The work in [7] adopted five features (i.e., frequency error, synchronization correlation, I/Q origin offset, magnitude error, and phase error) extracted from IEEE 802.11 frame signal to distinguish different NICs. The experiment results from an indoor wireless test-bed environment demonstrated that PARADIS could differentiate more than 130 NICs with an accuracy greater than 99%. Additionally, the authors in [12] exploited the non-linearity characteristic in the digital-to-analogue converter for device authentication and reported an authentication accuracy of 60% in their simulation with 100 devices and a signal-to-noise ratio (SNR) of 30 dB. The CFO was studied in [9] to distinguish 30 Xbee devices, and the experimental results reported an authentication accuracy of 95%. In addition, I/Q imbalance was investigated in [13] to distinguish four Zigbee devices, and an authentication accuracy of 100% was reported. In recent years, the authors in [5] explored a new feature, named visibility graph, of wireless signals and experimentally demonstrated that it could enhance the RFFA accuracy by being involved with the five features proposed in [7]. Furthermore, the authors in [6] proposed a new feature, named fractal dimension, of wireless signals and also theoretically analyzed and experimentally evaluated its effectiveness in RFFA. It can be seen from the literature that exploring new features has been an appealing research field to enhance the RFFA accuracy. Except for the above hand-draft feature-based RFFA, there is also another approach that utilizes the deep learning technique to automatically extract the “deep” fingerprint. In such an approach, the authors in [14] explored the device imperfections of controller area networks and found that, by applying the deep learning technique, the achieved RFFA accuracy increased from 92% to 96%. Moreover, the authors in [15,16] adopted convolutional neural networks to reduce the training complexity in RFFA and reported that the computation resources can be greatly reduced by using the CNN without affecting the accuracy. Towards this direction, the authors in [17] proposed to combine the signal samples from different receivers, which can reduce the required complexity in the neural networks, since it can obtain a benefit to accuracy from the data augmentation.

### 2.2. Traffic Anomaly Detection

Our considered security threat in MFS-based RFFA arises from the fact that an attacker may inject one forged frame into valid traffic, which compromises the MFS-based RFFA if such injected is not detected. Note that such an injection causes a traffic anomaly, and there exist two types of solutions in the literature for traffic anomaly detection, as listed in Table 3.

The first type of solution is detection-based, which aims to detect the appearance of anomalous traffic. The basic idea is to utilize the well-defined relationship between different frames: once a forged frame is injected, the relationship is corrupted and, thus, can be detected. The most widely studied inter-frame relationship is the sequence number [18,19]. Sequence number is a 16-bit sequence control field starting at 0, which is then incremented by one for each non-fragmented frame. Thus, a forged frame signal causes non-continuity of the sequence number (here, it is assumed that an attacker cannot prevent the communication between a valid transceiver pair). However, the sequence number is easy to predict and forge by the attacker using soft wireless card or by techniques proposed in [20]; in addition, the sequence number is not available for control and management frames. Except for the sequence number, the relationships, including received signal strength [21], arrival time [22], and channel response [23], are also studied in previous research. Although these anomaly detection techniques can provide lightweight detection of traffic anomaly, they cannot guarantee normal communication when such attacks occur. In other words, the receiver can only know the presence of a traffic anomaly but cannot locate the injected frame(s).

The second type of solution is prevention-based, which aims to detect the injected frame and then discard it to guarantee normal communication, even when the attack occurs. The most commonly utilized prevention-based solutions include the digital signature [24] and hash message authentication code (HMAC) [25]. The basic idea is that attackers do not know the key and, thus, cannot generate the correct digital signature or HAMC. However, such prevention-based solutions usually require pre-shared key between the transceiver. Accordingly, for scenarios where establishing the pre-shared key between the transceiver is too difficult or unavailable, we do not follow the prevention-based solutions in this paper.

## 3. System Model & Security Threat

### 3.1. Preliminary Knowledge to CSMA/CA

In the CSMA/CA protocol, following Section 5.1.4 in [26], devices initially synchronize with the network coordinator using beacon signals. A general transmission mechanism through a multiple-access channel was introduced in [26] as follows. When a device wants to transmit data, it senses the channel to check for ongoing activity. If the channel is busy, it continues sensing until the channel becomes idle in the next time slot. Next, the device waits for a short additional period called the distributed inter-frame spacing (DIFS), prioritizing frames with higher priority, such as real-time or urgent data. Once the DIFS expires and the channel remains idle, the device generates a random Backoff interval before transmitting. This random Backoff mechanism prevents multiple devices from transmitting simultaneously, thus avoiding collisions. For each time slot, the Backoff time counter is decremented by 1 as long as the channel is sensed idle, stopped when a transmission is detected on the channel, and reactivated when the channel is sensed idle again for more than a DIFS. When the Backoff time counter reaches zero and the channel is still idle, the device begins data transmission. Following this protocol ensures efficient communication among devices, reducing collision risks and optimizing data transfer within the network.

### 3.2. System Model

**Figure 2:**
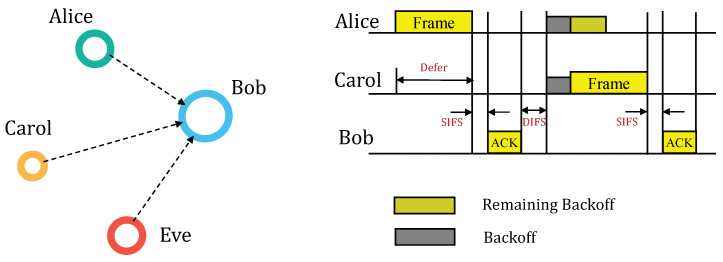
Communication model, where Alice and Carol communicate with Bob through a CSMA/CA channel. Eve aims to impersonate Alice to deceive Bob.

We consider the communication model depicted in Figure 2, where Alice and Carol act as transmitters, while Bob acts as the receiver. In this model, both Alice and Carol utilize the CSMA/CA protocol to access the channel. Bob responds with an ACK frame when he has successfully received a frame. Additionally present in the communication model is an impersonation attacker named Eve. Eve possesses knowledge about Alice, including her MAC address, IP address, and communication protocol. Eve’s objective is to transmit forged frames by impersonating Alice, with the intention of pursuing invalid interests.

To prevent the impersonation attack, when Bob has received *M* (M>1) frames claimed to be from Alice, Bob employs the MFS-based RFFA to determine whether the received *M* frames originate from Alice or Eve. We denote the *M* received frames signal by a matrix $Y=\left[y_{1},y_{2},\ldots ,y_{M}\right]$, where each element correspond to a frame signal. Bob first estimates the desired feature, denoted by ${\hat{f}}_{m\left(1⩽m⩽M\right)}\in R^{n}$, from each received frame signal $y_{m}$. The *M* feature estimates constitute a feature estimate vector $\hat{f}={\left[{\hat{f}}_{1},{\hat{f}}_{2},\ldots ,{\hat{f}}_{M}\right]}^{T}$. Furthermore, we consider that Bob knows a reference feature of Alice, denoted as *f*. The MFS-based RFFA scheme can be formulated as the following binary hypothesis test:

(1) $$\left\{\begin{array}{cc} H_{0}: & |\alpha \;\cdot \;\hat{f}-f|⩽\delta \\ H_{1}: & |\alpha \;\cdot \;\hat{f}-f|>\delta \end{array}\right.,$$

where $\alpha =\left[\alpha_{1},\alpha_{2},\ldots ,\alpha_{n}\right]$ represents the weight of different feature estimates, and δ represents the decision threshold. If $H_{0}$ is accepted, it implies that the received multi-frame signal Y originates from Alice. Conversely, if $H_{1}$ is accepted, it indicates that the received multi-frame signal Y originates from Eve, thus implying an impersonation attack.

### 3.3. Security Threat

**Figure 3:**
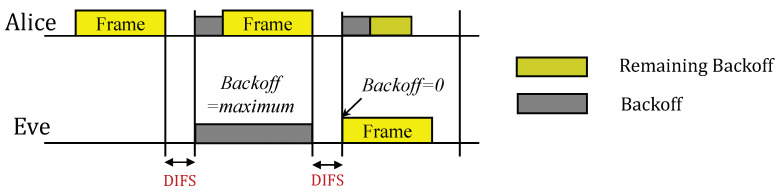
Arbitrary injection of a forged frame into valid traffic by Eve through modifying the Backoff time.

In CSMA/CA, Eve can inject a forged frame into valid traffic by modifying its Backoff time. As shown in Figure 3, Eve can arbitrarily activate injection by setting the Backoff time to zero, or deactivate injection by setting the Backoff time to the maximum value. This strategic control allows Eve to manipulate the channel access and, thus, the forged frame injection opportunity and ratio in valid traffic. As a result, the forged frame is blended with legitimate frames in the MFS-based RFFA.

**Figure 4:**
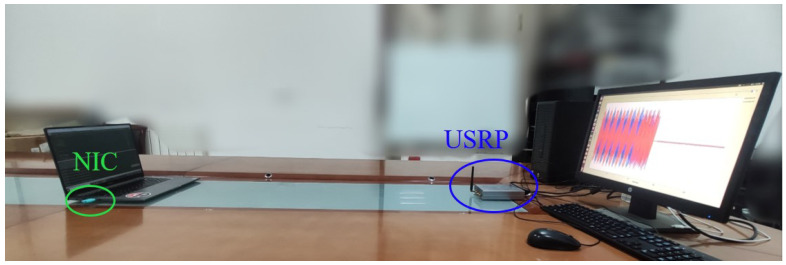
Experimental deployment.

To demonstrate the impact of the aforementioned forged frame injection on the MFS-based RFFA, we conducted a Proof-of-Concept experiment to measure the averaged feature estimate with multiple frames in the presence and absence of the aforementioned injection. For this purpose, we first developed a frame signal collection platform, as shown in Figure 4, in which we utilized a universal software radio peripheral (USRP) as the receiver to collect frames signal by setting the Network Interface Card (NIC) as the transmitter. Next, $10^{4}$ frame signals from two NICs, which represent Alice and Eve, were alternately sampled. We estimated, normalized, and recorded the CFO for each collected frame. Subsequently, we combined these CFO estimates to form a group of *M* samples comprising $M_{e}$ elements randomly originating from Eve and the remaining $M-M_{e}$ elements randomly originating from Alice. We then calculated the average CFO estimate for each group type by setting $\alpha =\underset{M}{\underset{︸}{\left[1,1,\ldots ,1\right]}}$.

**Figure 5:**
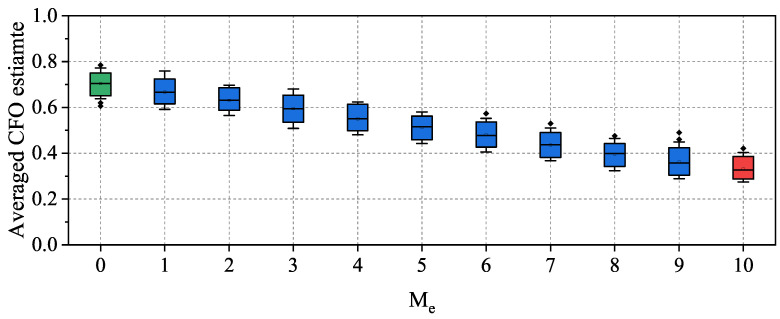
Box plots of averaged CFO estimates for different group classes, with the size M=10 and $M_{e}$ varying from 0 to 10. We show the 5th and 95th percentiles.

To analyze the statistical distribution in the averaged CFO estimate, we set M=10, and $M_{e}$ varied from 0 to 10 to form 11 group classes. For each group class, we generated $10^{3}$ samples, calculated the corresponding averaged CFO estimate of each sample, and used box plots, as illustrated in Figure 5, to represent the statistical characteristics of the averaged CFO estimate. The results in Figure 5 show that the averaged CFO estimate for classes $M_{e}=0$ and $M_{e}=10$ (green and red) exhibited the largest distinction, indicating that, if we can ensure all of the 10 frames originate from the same transmitter, the MFS-based RFFA can achieve the highest accuracy. However, as $M_{e}$ decreased from nine to one, the distribution of the averaged CFO estimate gradually became more and more similar to that of $M_{e}=0$. In particular, when $M_{e}=1$, more than half of the averaged CFO estimate overlapped with that of $M_{e}=0$. This suggests that, when Eve injects only one forged frame into valid traffic, the forged frame is much more likely to be accepted by the MFS-based RFFA. Overall, the results presented in Figure 5 demonstrate the severity of the identified injection attack in the MFS-based RFFA.

## 4. IfrPS Design and IfrPS-Aided, MFS-Based RFFA Scheme

In this section, we propose the IfrPS design and the IfrPS-aided, MFS-based RFFA scheme. At the end, we give a brief discussion of the security properties of our propositions.

### 4.1. IfrPS Design

**Figure 6:**
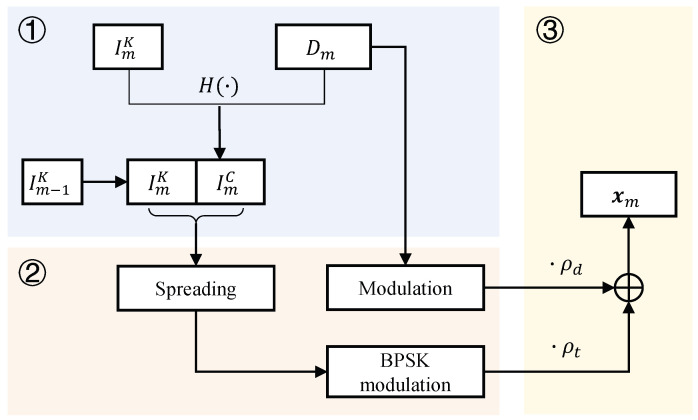
Flow diagram of the IfrPS design involving three steps ①, ② and ③. $D_{m}$ is the frame data to be transmitted, $x_{m}$ is the IfrPS obtained from $D_{m}$ and unique information $\left(I_{m}^{K},I_{m}^{C}\right)$.

The rationale behind the designed IfrPS is to associate each transmitted frame with unique information that cannot be forged by attackers. To this end, the transmitter attaches an HMAC to each transmitted frame signal and then discloses the key in the next transmitted frame signal (see the flow diagram of the IfrPS design illustrated in Figure 6). To elaborate further, we summarize the IfrPS design in three key steps.

First, we generate the unique information that needs to be attached in each transmitted frame signal. Let us denote the message data of the *m*-th frame by $D_{m}$, where *m* is interpreted as the frame index. It is worth noting that the re-transmitted frame is considered to have the same index *m*. Then, we can denote the unique information of the *m*-th frame by $\left(I_{m}^{K},I_{m}^{C}\right)$. Here, $I_{m}^{K}$ satisfies

(2) $$I_{m-1}^{C}=H\left(D_{m-1},I_{m}^{K}\right),$$

and $I_{m}^{C}$ is obtained by

(3) $$I_{m}^{C}=H\left(D_{m},I_{m+1}^{K}\right),$$

where $H\left(\cdot \right)$ represents the hash function. Note that, for each value of *m*, the transmitter randomly generates $I_{m}^{K}$, and $I_{m+j}^{K}$ and $I_{m+j}^{K}$ are independent of each other when i≠j. Based on the above, the receiver can detect whether two received frames, with signals $y_{m-1}$ and $y_{m}$, originate from the same transmitter by calculating whether the demodulated unique information and message satisfy Equation (2).

Second, we convert the unique information $\left(I_{m}^{K},I_{m}^{C}\right)$ into symbols before attaching it to the transmitted frame signal, as shown in Figure 6. Since the bit sizes of $I_{m}^{K}$ and $I_{m}^{C}$ are, at most, 128, which is a number less than the frame length in most applications, we spread the unique information $\left(I_{m}^{K},I_{m}^{C}\right)$ to match the frame length. To this end, we use the spreading code, denoted by $s=\left[s_{1},s_{2},\ldots ,s_{N/N_{I}}\right]$, with each element as aj or −aj (*j* is the complex symbol), where *N* represents the frame length and $N_{I}$ represents the bit size of $I_{m}^{K}$ and $I_{m}^{C}$. To simplify the description, we assume that *N* is a multiple of $N_{I}$. Let us denote the converted symbols $I_{m}^{K}$ and $I_{m}^{C}$ by $t_{m}^{K}$ and $t_{m}^{C}$, respectively. $t_{m}^{K}$ and $t_{m}^{C}$ are given by

(4) $$\left\{ \begin{array}{ccc} t_{m}^{K}\left[n\right] & = & I_{m}^{K}\left[n|\left(N/N_{I}\right)\right]\cdot s\left[n\;\mathrm{mod}\;\left(N/N_{I}\right)\right]\\ t_{m}^{C}\left[n\right] & = & I_{m}^{C}\left[n|\left(N/N_{I}\right)\right]\cdot s\left[n\;\mathrm{mod}\;\left(N/N_{I}\right)\right] \end{array},\right.$$

where “|” and “mod” are the symbols for division and modulus, respectively.

**Figure 7:**
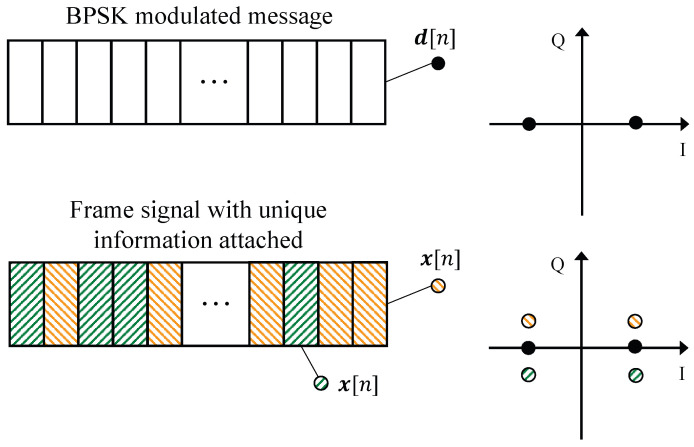
An illustrative example of attaching unique information into the frame signal where the message is modulated using BPSK.

Third, we attach the converted BPSK symbols of $\left(I_{m}^{K},I_{m}^{C}\right)$ into the modulated frame symbols. Let us denote the *m*-th frame without unique information being attached by ***d**_m_* = [*d*_*m*,1_, *d*_*m*2_,…, *d*_*m*,*n*_], and its version with unique information being attached by ***x**_m_*, Here, ***x**_m_* is given by

(5) $$\left\{ \begin{array}{cc} x_{m}[n]=\rho_{d}\cdot d_{m}[n]+\rho_{t}\cdot t_{m}^{K}[n], & n<N/2\\ x_{m}[n]=\rho_{d}\cdot d_{m}[n-\frac{N}{2}]+\rho_{t}\cdot t_{m}^{C}[n-\frac{N}{2}], & n⩾N/2 \end{array}.\right.$$

where *ρ_d_* and *ρ_t_* represent the power allocation for the message and unique information, respectively. Due to power constraint, we have $\rho_{d}^{2}+\rho_{t}^{2}=1$. To provide readers with a clearer understanding of the attaching method, we also present, in Figure 7, an illustrative example of the message being modulated with BPSK.

### 4.2. IfrPS-Aided, MFS-Based RFFA Scheme

Considering that the transmitter has sequential frames for transmission, denoted by $X=\left[x_{1},x_{2},x_{3},\ldots \right]$, and taking into account the retransmission mechanism at the MAC layer, we assume that all these frames can be successfully received and demodulated by the receiver, denoted by $Y=\left[y_{1},y_{2},y_{3},\ldots \right]$. However, each of the received frames may be forged and injected. In the IfrPS-aided, MFS-based RFFA scheme, the receiver needs to select the frames in Y that originate from the same transmitter as the first frame $y_{1}$ and construct a selected frames set denoted as $Y_{s}$. The receiver then inputs $Y_{s}$ into Equation (1) to obtain the authentication result of the first frame $y_{1}$. Similarly, for authenticating $y_{m}$, the receiver selects frames from $Y-\left\{y_{1},\ldots ,y_{m-1}\right\}$ and constructs the corresponding $Y_{s}$. We summarize the procedure for leveraging the property of IfrPS to obtain $Y_{s}$ for the authentication of $y_{1}$ by using an *M*-frame signal as follows. This method can be extended to the authentication of other frames.

$Y_{s}$ is initialized as $Y_{s}=\left\{y_{1}\right\}$. Frames in Y are, in turn, examined to be appended to $Y_{s}$ or not. We use $y_{1}$ and $y_{2}$ as an example to explain how to examine the IfrPS relationship between two consecutively frames. Note that each entry of $y_{1}$ and $y_{2}$ is given by

(6) $$\begin{array}{c} y_{m,n}=h_{m,n}\cdot x_{m,n}+w_{m,n},\;m\in \left\{1,2\right\} \end{array}$$

where $h_{m,n}∼N\left(0,\frac{1}{2}\right)$ represents the channel fading, and $w_{m,n}∼N\left(0,\sigma_{w}^{2}\right)$ represents the Gaussian noise. We assume block fading, so $h_{m,n}$ remains constant for the same value of *m* and varies independently across different values of *m*. Similarly, $w_{m,n}$ varies independently across different values of *m* and *n*. To extract the unique information attached in $y_{m}$, the receiver first equalizes $y_{m}$ and demodulates the message dm. Then, it demodulates the unique information using

(7) $$\begin{array}{c} {\hat{t}}_{m}=\frac{h_{m}^{H}}{{\left|h_{m}\right|}^{2}}\cdot y_{m}-d_{m}, \end{array}$$

where we assume accurate estimation of $h_{m}$ and decoding of $d_{m}$. Based on the obtained ${\hat{t}}_{m}$ from Equation (7), the receiver can obtain the unique information through BPSK demodulation and de-spreading. Let ${\hat{I}}_{m}^{C}$ and ${\hat{I}}_{m}^{K}$ represent the estimated HMAC and key, respectively. The receiver can determine whether $y_{1}$ and $y_{2}$ originate from the same transmitter using the following binary hypothesis test:

(8) $$\begin{array}{c} \left\{\begin{array}{cc} H_{0}: & D\left[{\hat{I}}_{1}^{C},H\left(D_{1},{\hat{I}}_{2}^{K}\right)\right]=0\\ H_{1}: & D\left[{\hat{I}}_{1}^{C},H\left(D_{1},{\hat{I}}_{2}^{K}\right)\right]>0 \end{array}\right., \end{array}$$

where D represents the code distance. If $H_{0}$ is accepted, the receiver appends $y_{2}$ to $Y_{s}$; otherwise, $y_{2}$ is not appended to $Y_{s}$. The receiver iteratively examines the last element in $Y_{s}$ and the first element in $Y-Y_{s}$, until either the size of $Y_{s}$ is *M* or the pair of frames to be examined has already been examined.

Remarks: In our proposed IfrPS-aided, MFS-based RFFA scheme, we focus on authenticating the first frame $y_{1}$. This differs from previous MFS-based RFFA schemes where the authentication result is used for all frame signals. This is because we cannot ensure whether the previous frame originates from the same transmitter by testing the IfrPS, as Eve, with significant computational resources, can deduce $I_{m+1}^{K}$ by listening to $I_{m}^{C}$ and $D_{m}$ (see the detailed discussion in the previous subsection).

### 4.3. Security Property

In this subsection, we discuss the security property of the designed IfrPS, to demonstrate its ability to counter forged frame injection in MFS-based RFFA.

**Figure 8:**
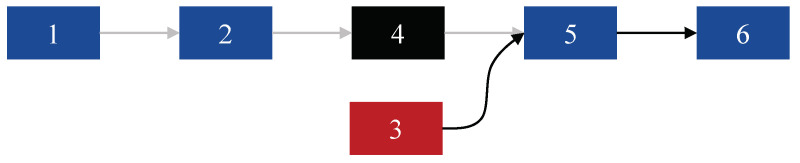
Forged frame injection in forming the MFS, where the forged frame cracks the inter-frame relationship between the 4th and 5th frames.

In MFS-based RFFA, where one forged frame is injected into valid traffic, there are two favorable cases for Eve in constructing the MFS. The first case occurs when the forged frame blends in with past valid frames, while the second case occurs when the forged frame blends in with the following valid frames. We have found that the proposed IfrPS design cannot prevent the first case, but it can effectively prevent the second case. Refer to Figure 8 for an illustrative explanation, where the marked numbers represent the frame index. In the following discussion, we analyze the security of the IfrPS design against these two cases separately.

The injected frame may blend in with the following two legitimate frames for MFS-based RFFA. This can be achieved if the conveyed $I^{C}$ in the third frame matches the $I^{K}$ conveyed in the fourth frame. However, this scenario cannot be achieved, since Eve has to transmit the third frame before the fourth frame. It is important to note that, if the third frame is transmitted after the fourth frame, the receiver will discard the third frame due to the incorrect sequence number. Thus, Eve cannot obtain any knowledge about the $I^{K}$ conveyed in the fourth frame to crack it. In other words, Eve has no way to generate the correct $I^{C}$ that should conveyed by the third frame for a successful injection.

Overall, we observe that the IfrPS design can prevent the injected frame from blending in with the following valid frames in MFS-based RFFA. Therefore, we deduce that our proposed IfrPS-aided, MFS-based RFFA scheme is secure, since the receiver explores the following frames to form the MFS for each received frame.

## 5. Efficiency

**Figure 9:**
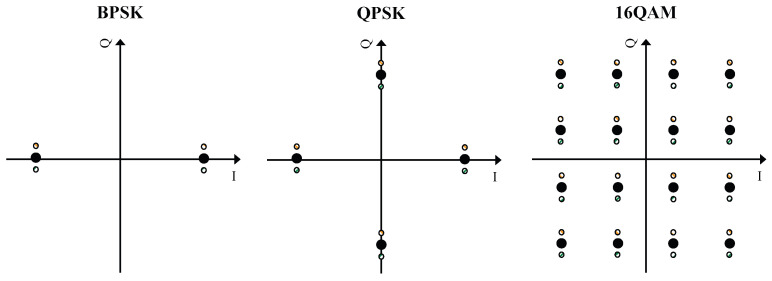
Constellation of superimposed symbols at the physical layer for the BPSK, QPSK, and 16QAM, respectively.

Since the designed IfrPS requires a portion of transmission power to convey the unique information, the message demodulation BER will inevitably be affected. We measured the efficiency of the IfrPS design by evaluating its impact on message demodulation error. To this end, we considered the BPSK, QPSK, and 16QAM, with the constellation with IfrPS design shown in Figure 9, and performed both theoretical analysis and numerical evaluation towards these three modulation systems to assess the efficiency of the IfrPS design.

With perfect channel estimation, the *n*-th received frame signal after channel compensation, denoted by $y_{n}^{c}$, can be expressed as

(9) $$y_{n}^{c}=\rho_{d}\cdot d_{n}+\rho_{t}\cdot t_{n}+\frac{1}{|h_{n}|}\cdot w_{n},$$

where we define $\frac{E_{s}}{E_{n}}=\frac{E\left(d_{n}\cdot d_{n}^{H}\right)}{E\left(w_{n}\cdot w_{n}^{H}\right)}=\frac{E\left(t_{n}\cdot t_{n}^{H}\right)}{E\left(w_{n}\cdot w_{n}^{H}\right)}$ to denote the transmission symbol-to-noise power ratio. On the basis, we have the transmission bit-to-noise power ratio of $\frac{E_{b}}{E_{n}}=\frac{E_{s}}{E_{n}}$ for BPSK, $\frac{E_{b}}{E_{n}}=\frac{1}{2}\cdot \frac{E_{s}}{E_{n}}$ for QPSK, and $\frac{E_{b}}{E_{n}}=\frac{1}{4}\cdot \frac{E_{s}}{E_{n}}$ for 16QAM. Let us denote the message demodulation BER of the IfrPS for BPSK, QPSK, and 16QAM systems by $P_{IfrPS,b}^{BPSK}$, $P_{IfrPS,b}^{QPSK}$, and $P_{IfrPS,b}^{16-QAM}$, respectively. By considering the Gray code mapping (refer to [27]), we can derive $P_{IfrPS,b}^{BPSK}$, and approximate $P_{IfrPS,b}^{QPSK}$ and $P_{IfrPS,b}^{16-QAM}$ by

(10) $$\begin{array}{c} P_{IfrPS,b}^{BPSK}=\frac{1}{2}\cdot \left(1-\sqrt{\frac{\rho_{s}^{2}E_{b}}{\rho_{s}^{2}E_{b}+E_{n}}}\right), \end{array}$$

(11) $$\begin{array}{c} P_{IfrPS,b}^{QPSK}\approx \frac{1}{2}-\frac{1}{4}\left[\sqrt{\frac{{\left(\rho_{s}+\rho_{t}\right)}^{2}E_{b}}{{\left(\rho_{s}+\rho_{t}\right)}^{2}E_{b}+E_{n}}}+\sqrt{\frac{{\left(\rho_{s}-\rho_{t}\right)}^{2}E_{b}}{{\left(\rho_{s}-\rho_{t}\right)}^{2}E_{b}+E_{n}}}\right], \end{array}$$

and

(12) $$\begin{array}{cc} P_{IfrPS,b}^{16QAM}\approx \\ \; & =\frac{3}{8}-\frac{3}{32}\left[\sqrt{\frac{{\left(\rho_{s}+\rho_{t}\right)}^{2}E_{b}}{{\left(\rho_{s}+\rho_{t}\right)}^{2}E_{b}+E_{n}}}+\sqrt{\frac{{\left(\rho_{s}-\rho_{t}\right)}^{2}E_{b}}{{\left(\rho_{s}-\rho_{t}\right)}^{2}E_{b}+E_{n}}}\right]\\ \; & -\frac{3}{16}\sqrt{\frac{E_{b}}{E_{b}+E_{n}}}, \end{array}$$

respectively.

**Figure 10:**
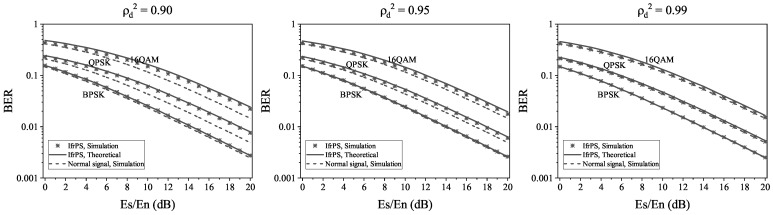
Theoretical and numerical results of the message demodulation BER of the IfrPS, where the message demodulation BER of the normal signal is also plotted for comparison.

We present both theoretical and numerical results for the message demodulation BER of IfrPS in the presence of BPSK, QPSK, and 16QAM modulations, as well as the BER of the normal signal (without IfrPS), for comparison. Figure 10 illustrates these results.

The first observation from the figure is that the theoretical and numerical results for the message demodulation BER of IfrPS exhibit a small discrepancy. This suggests that our theoretical analysis serves as a reliable predictor for the message demodulation BER of IfrPS. The second observation is that the message demodulation BER of IfrPS is only slightly higher than that of the normal signal across various signal-to-noise ratio (SNR) levels. When $\rho_{d}^{2}=0.99$, the obtained BERs are nearly identical. This indicates that IfrPS introduces only a minor performance degradation in message demodulation, making it suitable for applications with stringent requirements on demodulation accuracy. The third observation is that the message demodulation BER of IfrPS is influenced by $\rho_{d}^{2}$, where a larger $\rho_{d}^{2}$ results in a higher BER. This implies that we can adapt the parameter $\rho_{d}^{2}$ to meet different requirements for message demodulation BER in practical applications. The fourth observation is that, under the same system parameters, the impact of IfrPS on the message demodulation BER varies across different modulation systems. For example, when $\rho_{d}^{2}=0.90$, the equivalent SNR degradation in message demodulation is approximately 0.45 dB for BPSK, whereas it is around 2 dB for 16QAM. This suggests that the effect of IfrPS on BER is less pronounced in low-order modulation systems.

In summary, the results in Figure 10 demonstrate the effectiveness of IfrPS, as the message demodulation BER is only slightly increased when $\rho_{d}^{2}$ is appropriately set. In the next section, we further explore the resulting accuracy gain in RFFA using the same $\rho_{d}^{2}$ setting for IfrPS.

## 6. Effectiveness

The ability to securely construct an MFS inevitably affects the accuracy of an MFS-based RFFA scheme. Thus, we evaluate the effectiveness of the IfrPS-aided, MFS-based RFFA scheme in terms of the resultant RFFA accuracy that can be achieved. To measure the effectiveness, we define two types of error as follows:

- *FRR:* FRR represents the ratio of valid samples that are incorrectly classified as invalid;
- *FAR:* FAR represents the ratio of invalid samples that are incorrectly classified as valid.

Note that these two types of error affect both the processes of IfrPS detection and RFFA at the receiver side, i.e., the Equations (1) and (8). In the context of IfrPS detection, a valid sample refers to a frame signal originating from the same transmitter as the previous frames, while an invalid sample refers to a frame signal originating from a different transmitter. In the context of RFFA, a valid sample refers to a frame signal originating from Alice, while an invalid sample refers to a frame signal originating from Eve. In the following, we first analyze and evaluate these two types of error for the IfrPS detection process and then, on that basis, analyze and evaluate these for the RFFA process.

### 6.1. FRR and FAR in IfrPS Detection

We derived the closed-form expression of the FRR and the numerical solution of the FAR in the IfrPS detection process.

**Theorem 1.**  *The FRR in IfrPS detection, denoted by $P_{FRR}^{IfrPS}$, is given by*

(13) $$\begin{array}{cc} \; & P_{FRR}^{IfrPS}=1-\int_{0}^{\infty }\int_{0}^{\infty }{\left[1-\frac{1}{2}erfc\left(h_{1}\sqrt{L}\rho_{t}\sqrt{\frac{E_{s}}{E_{n}}}\right)\right]}^{\frac{N}{2L}}\\ & \cdot {\left[1-\frac{1}{2}erfc\left(h_{2}\sqrt{L}\rho_{t}\sqrt{\frac{E_{s}}{E_{n}}}\right)\right]}^{\frac{N}{2L}}\cdot \exp\left(-h_{1}\right)\cdot \exp\left(-h_{2}\right)\\ \; & \cdot dh_{1}\cdot dh_{2} \end{array}$$

*and the FAR of IfrPS, denoted by $P_{FAR}^{IfrPS}$, is given by*

(14) $$\begin{array}{c} P_{FAR}^{IfrPS}={\left(\frac{1}{2}\right)}^{\frac{N}{2L}}. \end{array}$$

**Proof.**  For two consecutively received frame signals originating from the same transmitter, let us denote the channel fading coefficient of the first frame signal by $h_{1}$ and that of the second frame by signal $h_{2}$. We can express the probability that the unique information attached into these two frames are accurately demodulated by

(15) $$\begin{array}{c} P_{correct,1}={\left[1-\frac{1}{2}erfc\left(h_{1}\sqrt{L}\rho_{t}\sqrt{\frac{E_{s}}{E_{n}}}\right)\right]}^{\frac{N}{2L}}, \end{array}$$

and

(16) $$\begin{array}{c} P_{correct,2}={\left[1-\frac{1}{2}erfc\left(h_{2}\sqrt{L}\rho_{t}\sqrt{\frac{E_{s}}{E_{n}}}\right)\right]}^{\frac{N}{2L}}, \end{array}$$

respectively. Then, by integrating $P_{correct,1}$ and $P_{correct,1}$, we can obtain the probability that these two frames signal match with the demodulated unique information by

(17) $$\begin{array}{cc} P_{match,1,2}= & \int_{0}^{\infty }\int_{0}^{\infty }P_{correct,1}\cdot P_{correct,1}\cdot \exp\left(-h_{1}\right)\\ \; & \cdot \exp\left(-h_{2}\right)\cdot dh_{1}\cdot dh_{2}. \end{array}$$

Substituting $P_{FRR}^{IfrPS}=1-P_{match,1,2}$ into Equation (17), we can prove Theorem 1. □

**Figure 11:**
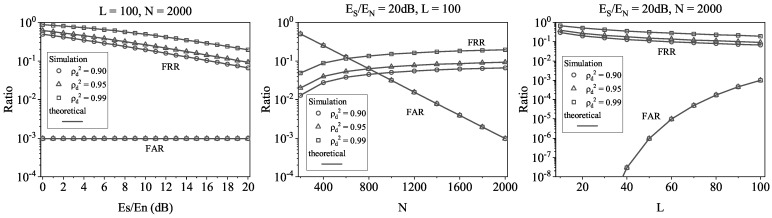
Theoretical and numerical results of FRR and FAR in the IfrPS detection process.

We plot the theoretical and numerical results of the FRR and FAR in IfrPS detection process in Figure 11. The first observation is that the theoretical results match the numerical results well, which indicates that our theoretical expressions can be used to predict the performance. The second observation is that the resultant FRR and FAR perform a trade-off relationship over *N* and *L*. This indicates that, in practical applications, the values of *N* and *L* need to be optimized to achieve the required FRR and FAR levels. The third observation is that the resultant FRR and FAR can be refined with a larger $E_{s}/E_{n}$ and a smaller $\rho_{d}^{2}$. This indicates that, for a communication with larger SNR and tolerance on message demodulation degradation, we can always obtain better performance in IfrPS detection.

Furthermore, to provide a visual presentation of the IfrPS detection performance, we calculate the expected sequence length of the detected MFS, denoted by *M*. Note that the calculation can be expressed by

(18) $$\begin{array}{c} M=\sum_{m=1}^{\infty }{\left(1-P_{FRR}^{IfrPS}\right)}^{m-1}\cdot P_{FRR}^{IfrPS}\cdot m. \end{array}$$

**Figure 12:**
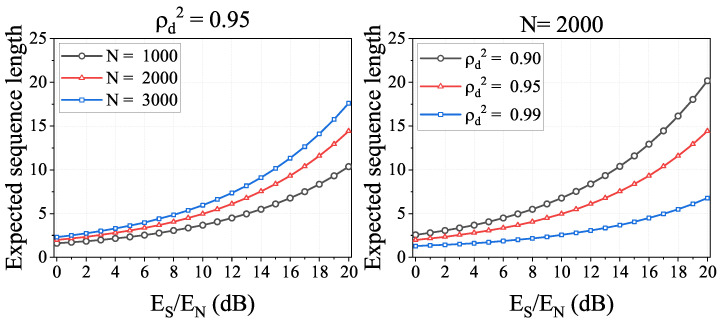

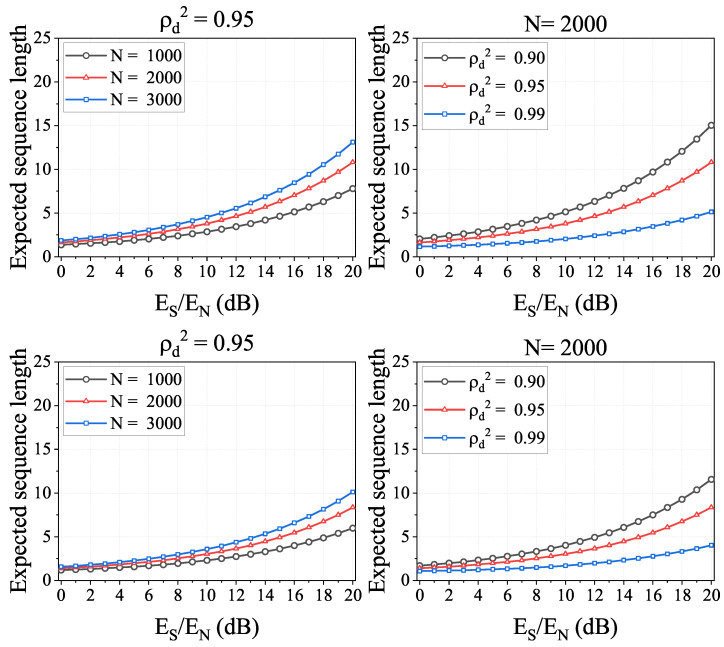
Simulation results of expected sequence length. Tested under the FAR of IfrPS set to be 0.01 (the top figures), 0.001 (the middle figures) and 0.0001 (the bottom figures).

Additionally, we plot the results in Figure 12. The first observation is that the expected sequence length increases over $E_{s}/E_{n}$ and decreases over $\rho_{d}^{2}$. This is easy to understand, since we have demonstrated above that the IfrPS detection performance is positive in relation to $E_{s}/E_{n}$ and negative to $\rho_{d}^{2}$. The second observation is that, for different levels of FAR in IfrPS detection, the obtained *M* has quite a significant value. For instance, when the FAR is fixed at 0.0001, i.e., the attacker can only compromise the IfrPS detection with the probability of 0.0001, the obtained *M* is more than 10 when N=2000 and $\rho_{d}^{2}=0.90$. Since such parameters are easy to satisfy in practical applications, whereas the corresponding parameter ($\rho_{d}^{2}=0.90$) results in only about 2 dB degradation to message demodulation, the results in Figure 12 demonstrate the potential of enhancing the RFFA by adopting the IfrPS design and using the MFS-based approach.

### 6.2. FRR and FAR in RFFA

To quantify the FRR and FAR in RFFA, we used the CFO as the authentication feature as a case study. Following [28,29], we know that the CFO estimates follow the Gaussian distribution $N\left(\epsilon ,\frac{1}{4\pi^{2}L_{s}^{3}\left(N_{s}-1\right)\gamma }\right)$, where ϵ represents the expectation of CFO estimate, γ represents the received SNR, and $L_{s}$ and $N_{s}$ are two parameters in CFO estimation following $L_{s}\cdot N_{s}=N$. In this study, we fixed $L_{s}=2$ and calculated $N_{s}$ by $N_{s}=N/L_{s}$. Moreover, we can deduce that the averaged CFO estimate with *M* frames follows the distribution $N\left(0,\frac{1}{4\pi^{2}L_{s}^{3}\left(N_{s}-1\right)\bar{\gamma }}\right)$, where $\bar{\gamma }=\frac{1}{M}\cdot \sum_{m=1}^{M}\gamma_{m}$.

We consider that the CFO of the randomly selected attacker follows the uniform distribution $U\left(0,R\right)$, where *R* denotes the allowable CFO range, which we set to be 0.02π [28]. Thus, we can express the FAR in RFFA by

(19) $$\begin{array}{c} P_{FAR}^{SFS}=\frac{2\delta }{0.02\pi } \end{array}$$

for the SFS-based RFFA, and by

(20) $$\begin{array}{c} P_{FAR}^{MFS}=P_{FAR}^{IfrPS}+\left(1-P_{FAR}^{IfrPS}\right)\cdot \frac{2\delta }{0.02\pi } \end{array}$$

for the MFS-based RFFA.

**Theorem 2.**  *With the threshold δ, the FRR in the IfrPS-aided, MFS-based RFFA can be expressed by*

(21) $$\begin{array}{ccc} P_{FRR} & = & \sum_{m=1}^{\infty }p\left(m\right)\cdot P_{FRR}\left(m\right)\\ \; & = & \sum_{m=1}^{\infty }{\left(1-P_{FRR}^{IfrPS}\right)}^{m-1}\cdot P_{FRR}^{IfrPS}\cdot P_{FRR}\left(m\right), \end{array}$$

*where $P_{FRR}^{IfrPS}$ is given in Theorem 1, and $P_{FRR}\left(m\right)$ follows*

(22) $$\begin{array}{c} P_{FRR}\left(m\right)=erfc\left(\frac{\delta }{2\pi L_{s}^{1.5}\sqrt{\left(N_{s}-1\right)\bar{\gamma }}}\right). \end{array}$$

**Figure 13:**
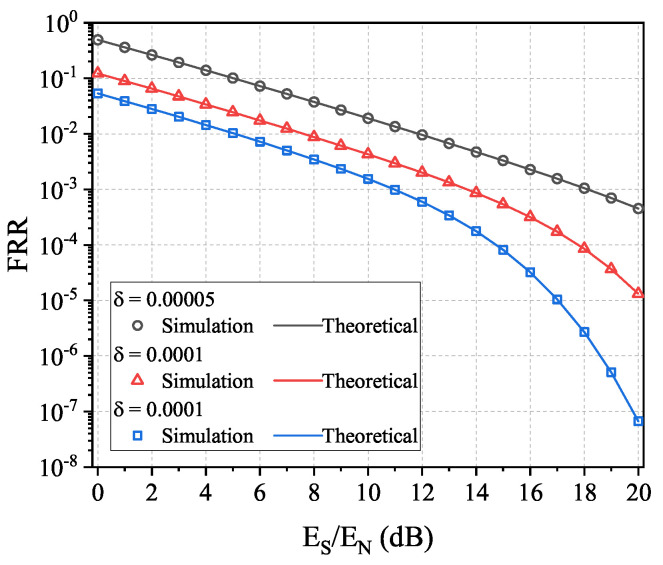
Theoretical and simulated FRR in the IfrPS-based multi-frame signal-based RFFA. Tested under N=2000, L=100, $\rho_{s}^{2}=0.90$.

To prove the above theorem, we illustrate, in Figure 13, both the theoretical and numerical FRR in RFFA, where we fix N=2000, L=100, and $\rho_{d}^{2}=0.95$. It can be observed from Figure 13 that the theoretical results of FRR in RFFA match the numerical results well. This indicates that our theoretical result can be used for predicting the FRR in IfrPS-aided, MFS-based RFFA. From Equations (19) and (20), we know that using a smaller threshold in IfrPS-aided, MFS-based RFFA system can achieve the same FAR as that using a smaller threshold in the SFS-based RFFA system, which indicates that we need to use a smaller threshold to ensure a smaller FRR at the same FAR level in RFFA through the IfrPS-aided, MFS-based one than the SFS-based one.

**Lemma 1.**  *To ensure the same FAR level of RFFA in the IfrPS-aided, MFS-based RFFA as that in the SFS-based one, and minimize the achieved FRR, the transmitter needs to optimize L.*

**Figure 14:**
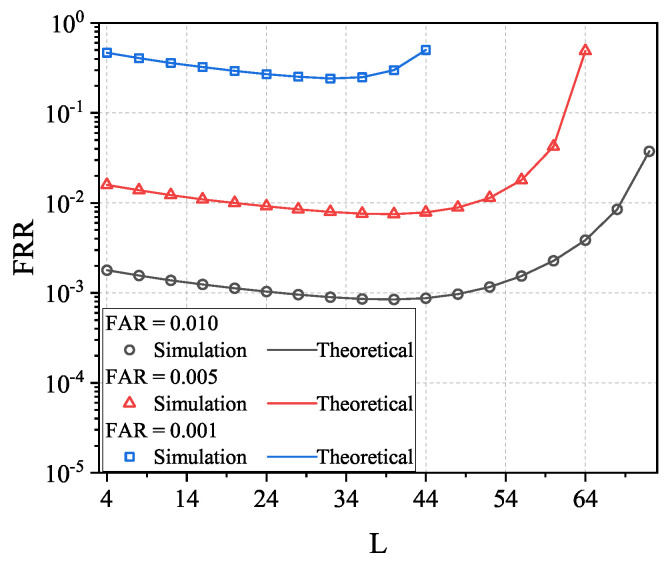
Theoretical and simulated FRR in the IfrPS-based multi-frame signal-based RFFA, with different FAR values. Tested under N=1000, $\rho_{d}^{2}=0.95$.

To prove the feasibility of using the above method for optimizing *L* and, thus, to reduce the FRR under the same FAR levels, we illustrate in Figure 14 the obtained FRR by searching the optimal *L*. Note that the optimal *L* is numerically searched using the FRR and FAR expressions in Equations (20) and (21). The first observation from Figure 14 is that the searched *L* is the optimal one since it leads to the minimal FRR for both the theoretical and numerical results. The second observation is that the relationship between FRR and *L* is the convex function and, thus, we can always search the optimal *L*.

**Figure 15:**
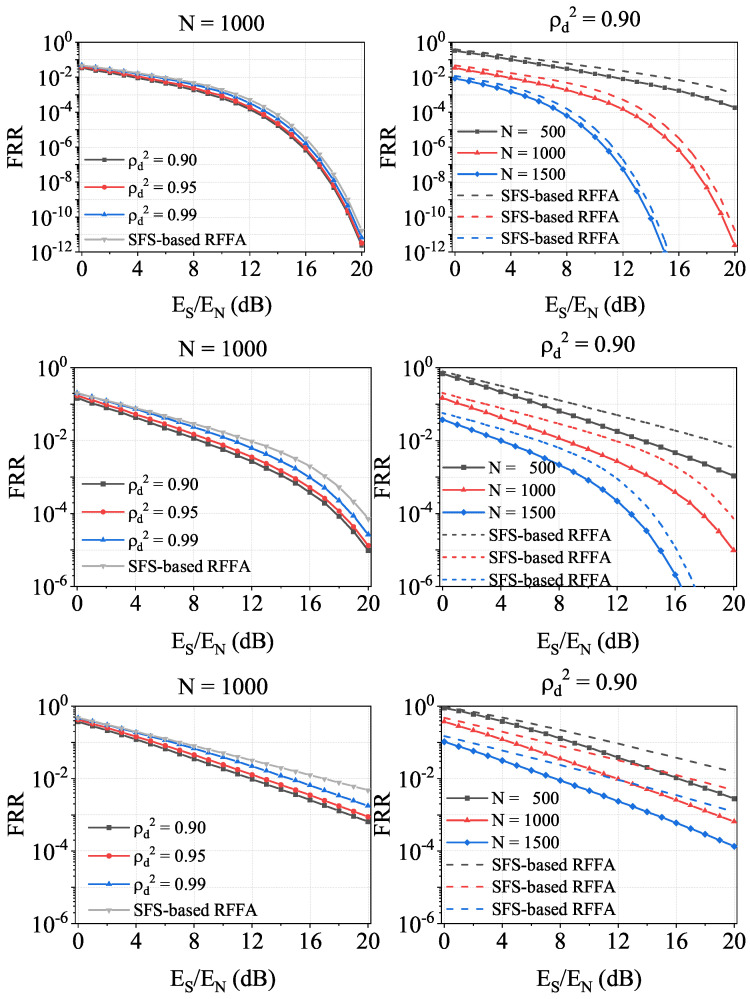
Numerical results of the minimal FRR achieved by the IfrPS-aided, MFS-based RFFA, tested under FAR levels of 0.01 (the top figures), 0.001 (the middle figures) and 0.0001 (the bottom figures).

Finally, in oder to demonstrate the FRR gain in IfrPS-aided, MFS-based RFFA over the MFS-based RFFA, we illustrate the numerical results under different levels of FAR in Figure 15. The first observation from Figure 15 is that we can always achieve a positive FRR gain. Furthermore, the FRR gain increases with a smaller $\rho_{d}^{2}$. The second observation from Figure 15 is that equivalent SNR gain is mainly related to $\rho_{s}^{2}$ rather than *N*. This is because using a larger *N* requires a larger *L* to ensure the FAR level, which inevitably limits the improvement in RFFA achieved by a larger *N*. The third observation from Figure 15 is that the equivalent SNR gain is about 5 dB when $\rho_{d}^{2}=0.90$, N=1500, and $E_{s}/E_{n}\;=\;20$ dB. Note that the corresponding equivalent SNR degradation to message demodulation is only 2 dB for 16QAM, 1 dB for QPSK, and 0.5 dB for BPSk. This demonstrates the effectiveness of the proposed IfrPS-aided, MFS-based RFFA scheme in securely improving the RFFA accuracy.

## 7. Conclusions

In this paper, we have identified a security threat associated with the MFS-based RFFA in CSMA/CA. To counter this security threat, we propose an IfrPS-aided, MFS-based RFFA scheme. We conducted a comprehensive study to evaluate the security, efficiency, and effectiveness of the proposed scheme and conducted simulations to evaluate its performance. We note that the proposed scheme can counter the identified security threat with no pre-shared key required between the transceiver, and can be applied to various communication systems while only causing minor impact on message demodulation. Overall, our contributions advance the field of RFFA techniques by, for the first time, highlighting a new but critical viewpoint of the security threat when utilizing a multi-frame signal for RFFA in the CSMA/CA system. Additionally, we provide a foundation for further research and development in this area to securely utilize the multi-frame signal in RFFA in the CSMA/CA system without requirements for pre-shared keys.

## Figures and Tables

**Table 1:** Summary of abbreviations.

Abbreviation	Full Name
MFS	Multi-frame signal
SFS	Single-frame signal
RFFA	Radio frequency fingerprint authentication
CSMA/CA	Carrier-sense multiple access with collision avoidance
IfrPS	Inter-frame-protected signal
I/Q	In-phase/quadrature
BPSK	Binary phase shift keying
QPSK	Quadrature phase shift keying
16QAM	16-quadrature amplitude modulation
CFO	Carrier frequency offset
FRR	False reject rate
FAR	False accept rate
SNR	Signal-to-noise rate
HMAC	Hash message authentication code
ACK	Acknowledgment
MAC	Medium access control
IP	Internal protocol

**Table 2:** Classification of researches on RFFA.

Class	Description	Reference
Hand-craft feature	Extracting features with artificially designed algorithms	[5,6,7,9,12,13]
Deep feature	Extracting features automatically using neural networks	[14,15,16,17]

**Table 3:** Classification of research on traffic anomaly detection.

Class	Description	Reference
Detection-based	Detect the appearance of anomalous traffic	[18,19,20,21,22,23]
Prevention-based	Detect the injected frame for discarding	[24,25]

## Data Availability

The data used can be obtained from the authors upon reasonable request.
